# Simulations on Simple Models of Connexin Hemichannels Indicate That Ca^2+^ Blocking Is Not a Pure Electrostatic Effect

**DOI:** 10.3390/membranes11050372

**Published:** 2021-05-20

**Authors:** Felipe Villanelo, Jorge Carrasco, Joaquin Jensen-Flores, Jose Antonio Garate, Tomas Perez-Acle

**Affiliations:** 1 Computational Biology Laboratory, Fundación Ciencia & Vida, Zañartu 1482, Ñuñoa, Santiago 7780132, Chile; felipe@dlab.cl (F.V.); j.carrasco@dlab.cl (J.C.); joaco.jensen@dlab.cl (J.J.-F.); 2 Facultad de Ingeniería y Tecnología, Universidad San Sebastián, Santiago 8420524, Chile; 3 Centro Interdisciplinario de Neurociencia de Valparaíso, Universidad de Valparaíso, Valparaíso 2360102, Chile; jose.garate@uv.cl

**Keywords:** connexin, hemichannel, calcium-binding, simulation

## Abstract

Connexin hemichannels allow the unspecific but regulated interchange of molecules from ions to second messenger and ATP, between the eukariotic cell and its extracellular space. The transport of ions and water through hemichannels is important for physiological functions and also in the progression of several pathological conditions. Extracellular Ca^2+^ concentration is one of the regulators that drives the channel to a closed state. However the relation between their functional and structural states is far for being totally understood. In this work, we modelled connexin hemichannels using simple systems based on a fixed array of carbon atoms and assess the Ca^2+^ regulation using molecular dynamics simulations. The two proposed mechanism described so far for calcium action were studied combined, e.g., an electrostatic effect and a pore stretching. Our results show that the addition of positive charge density inside the channel cannot stop the flow of potassium, chloride nor water. Only a pore stretching at the center of the pore can explain the channel blocking.

## 1. Introduction

Hemichannels (HC) are eukariotic protein channels that connects a cell with the extracellular space, allowing the interchange of a wide range of solutes, from ions to small peptides. An HC can bind to another HC in an adjacent cell to form a gap junction channel (GJC). HCs are composed of six subunits of the connexin protein. HCs and GJCs are involved in many biological process such as electrical synapse, immune responses, cell development and differentiation, among others.

Multiple mutations in connexins genes have been associated with several diseases in humans [1,2]. For example, the activity of HCs has been associated with the progression of diseases like cancer [3], cardiac ischemic injury [4], liver diseases [5], among others.

GJCs and HCs have complex mechanisms of regulation that include factors like pH [6], extracellular calcium [7] and voltage differences across the membrane [8]. Using these regulators, several functional states have been described mainly by electrophysiologic methods, e.g., open-state, closed-state and a range of intermediate states. But a key aspect that remains to be unsolved is the relation between these functional states and the structural conformation of the channels.

The most detailed structural information comes from the 3.5 Å resolution crystallographic model of human Cx26-GJC [9]. This model was initially identified as representative of an open-state channel, because the unobstructed path through the pore. However, several regions and amino acids were not resolved in this structure. Two different analysis had suggested that this model was not in an open-state, attending to the position of N-terminal helix (NTH), the lack of the initial methionine, not resolved in the original work and the lack of post-translational modifications (PTMs) [10,11].

But still the question around the closed-state structure and the transition from the open-state remains unsolved. Experiments have shown that the HC of several connexin isoforms undergoes conformational changes under different stimulus. Parahelix (PH) region of Cx46 can experiment changes in the pore diameter to values lower than 4 Å under loop-gating [12,13]. Similarly, atomic-force microscopy (AFM) of Cx26-HC [14] and Cx40-HC [15] have shown that extracellular calcium induces a pore narrowing in the extracellular region, with an estimated diameter of 5 Å [14]. Other evidences point to the involvement of NTH in conformational changes that generate a closed pore [16,17,18,19]

Subsequent crystallographic structures have been released, but the closed-state or the conformational changes that make possible the open-close transition remains elusive [20,21]. One interesting case is the recent resolution of the cryo-EM structure of Cx31.3 HC in an apparent closed state, but it is not clear if the rest of the connexin isoforms use the same mechanism [21].

Nevertheless, two structures had raised an interesting questioning to the conformational change hypothesis. The crystallographic structures of Cx26-GJC with and without bound calcium, are almost identical [22]. After MD relaxation and electrostatic surface calculations, the authors arrive to a bold conclusion: the positive-charged ring formed by the six bound calcium ions in the PH region of each HC is sufficient to block the passage of ions solely by the action of the electrostatic potential without any conformational change. Although subsequent publications have rebated this hypothesis [23,24] the possibility that the closed-state could be triggered only by an electrostatic effect and not by a physically-occluded pore, is appealing enough to explore systematically.

One difficulty to assess the open-close transition by a blocker like calcium is the multiplicity of factors involved, e.g., voltage difference across the membrane, nature of solvent particles, lipid dynamics, calcium charge and all possible conformational change in a channel with six subunits of 200 residues. So, we create very simple models based on previously used one [25], that allow us to eliminate some of the involved factors and focus on others. This simplified Cx26 HC model -a fixed carbon lattice with judiciously placed charges that reproduce the real surface potential- eliminate all the dynamics related to the channel itself and the membrane. This way we can focus on solvent movement and calcium properties. To robust our analysis we also add a possible stretching of the pore in the central region, but without the open-closed transition, just the possible closed state in the simple channel.

Our results show that the addition of a positive charge density inside the tube, mimicking a full calcium-bound HC, is not sufficient to completely inhibit ionic transport. Only with a very high and non-physiological charge densities we could block completely the potassium permeation, but not chloride nor water flux. On the other hand, a pore stretching at the center of the pore can diminish the solvent permeation to the point of total block, when the pore diameter is 5 Å.

## 2. Materials and Methods

### 2.1. Modelling

Akin our previous work in modelling Cx26-GJC [25], Cx26-HC models were defined as a neutral $sp^{2}$ carbons arrayed as a body-centered cubic network lattice with a spacing of 4 Å. These atoms are meant to separate both aqueous phases of the system, emulating a lipid bilayer. These atoms aren’t affected by the electric field or any other potential in the system. To simulate the HC, a straight pore communicating both aqueous phases was drilled in the middle of the $sp^{2}$ membrane, roughly following the Cx26-HC dimensions, with an inner diameter of 20 Å, measured as the center-to-center distance between opposite carbons atoms in the pore. Aiming at reproducing the inner electrostatic surface of the hCx26-HC structure, two rings of carbon atoms, one in the equivalent-to-extracellular side and the other in the equivalent-to-cytoplasmic side contain partial charges of −0.2e and +0.2e for each atom, respectively. The bound-calcium ions effect was included by the presence of positive charge densities with values that ranged from 0.0e to +0.6e at positions equivalent to calcium positions observed in the crystallographic model [22], these atoms are referred to as calcium-like particles (CaLP). The choosing of the exact magnitude of the charge for CaLPs is not trivial, because the others charges in the pore are fractional to match the fewer freedom degrees of the model [25], consequently we cannot just add a net charge of +2 to each CaLP. We choose to make a more systematic approach, adding charge to CaLPs in magnitudes that were multiples of 1, 2 and 3 of the other charges, generating models with three different CaLP charges, +0.2e, +0.4e and +0.6e, respectively. These systems were called X1, X2 and X3 and X0 for uncharged CaLP or calcium-free equivalent HC.

**Figure 1:**
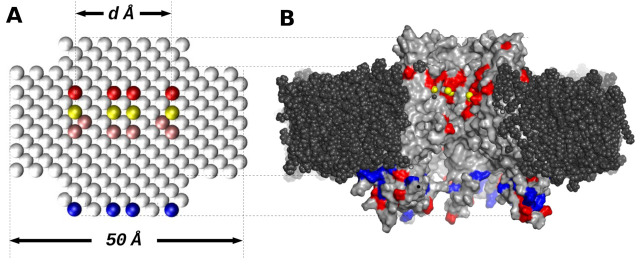
General scheme comparing the simple HC model (Panel A) with respect to the full-atom model of Cx26-HC embedded in a phospholipid bilayer (Panel B). (**A**) White spheres represent $sp^{2}$ carbon atoms; yellow spheres represent CaLPs; pink spheres represent the atoms subjected to radius alteration; red and blue spheres represent negative and positive charged dummy-carbons particles, respectively (see text for detail). (**B**) Membrane atoms are represented as black spheres; yellow spheres represent calcium atoms; grey surface is the HC protein surface, red and blue surface represent negative and positive residues. The upper region of both models is equivalent to the extracellular side.

The pore stretching effect was modelled through atoms at the pore center, with augmented $r_{min}$ parameters for the Lennard-Jones interaction. The original $r_{min}$ value of 4.0 Å for $sp^{2}$ carbon was increased to 8.0, 10.0, 12.0, 14.0 and 16.0 Å, generating 5 variants of these channels with different effective central diameters. These effective diameters, measured from water distribution, are 17, 13, 11, 9, 7 and 5 Å, for more details refer to the results section. As a reference, the equilibrated model obtained by Kwon et al. [10] have a minimal diameter of 12–14 Å after equilibration MD. We choose the central region of the pore to narrow because it is the approximate position of NTH in real HCs. Several authors have proposed this region as the responsible for the stretching of the pore in the closed-state HC [16,17,19]. It has been shown that the PH also undergoes changes upon voltage difference, narrowing the pore to value lower than 4 Å [12]. Moreover the PH region is the binding site for calcium ions, thus we kept the distances observed in the calcium-bound crystallographic model of Cx26-GJC [22]. In the latter, the center-to-center distance between opposing calcium is around 21 Å, similar to our models. A general depiction of the model is shown in Figure 1. A total of 24 models were generated, as a result from the combination of CalP charges and $r_{min}$ radiuses. All the models were placed in a box of 50 × 50 × 130 Å and solvated with explicit TIP3P water [26] and 150 mM potassium chloride using the software VMD v1.9 [27].

The all-atom Cx26-HC calcium-bound model is obtained from the crystallographic calcium-bound Cx26-GJC [22] with added missing regions from the previous Cx26-GJC structure [9], which was latter placed into a pre-equilibrated and solvated (TIP3P) POPC membrane, with box size dimensions of 150 × 150 × 130 Å.

### 2.2. Molecular Dynamics Simulations

The CHARMM36 forcefield [28] and the NAMDV2.13 software [29] were used to run all the MD simulations in this study; all systems were coupled to a thermal bath via Langevin dynamics, T = 298 K with a damping coefficient of 1 ps${}^{-1}$, at constant volume and number of particles. The Particle-mesh Ewald (PME) [3] method was utilized for long-range electrostatics within a tolerance of $1\times 10^{-6}$ and a grid-spacing of 1 Å; a cut-off distance of 12 Å was applied to real-space Ewald interactions and for van der Waals interactions. Multiple time steps were used with 1 fs for bonded interactions, 1 fs for short-range non-bonded interactions, and 2 fs for the full electrostatics evaluation using the r-RESPA method [30]. The SHAKE algorithm [31] was applied to constrain bond lengths to all hydrogen atoms. Periodic boundary conditions were employed in all dimensions.

Before production runs and for each model, an initial minimization of 500 steps was performed, followed by a short equilibration of 10 ns. Then, another step of equilibration was run for 100 ns. Afterwards, three independent production runs of 20 ns were carried out to collect data, applying the same external electric field described herein (see below). For the all-atom Cx26-HC calcium-bound simulation, after 50 ns of equilibration we run a production simulation of 10 ns, where the calcium ions were restrained into their positions. The others conditions of simulations were the same as previously described.

To simulate a voltage difference across the membrane, we used a constant electric field method [32]. Briefly, a constant positive electric field applied along the *z* axis exerts a force ($F_{ia}$) on each atom which follows:

(1) $$F_{ia}=q_{ia}E_{z}$$

where $q_{ia}$ stands for the atomic partial charge of atom *i*. The total potential $\Delta V_{z}$ across the periodic box exerted by the external electric simply follows the relation:

(2) $$\Delta V_{z}=-E_{z}L_{z}$$

where $L_{z}$ is the length of the periodic box along the *z* axis. In this work and for all productions runs the electric field applied result in a $\Delta V_{z}=500\;\mathrm{mV}$.

To evaluate what HC simple system is closer the calcium-bound all-atom model, in terms of electric activity, we evaluate the electrostatic potential in the z-slice where CaLP and calcium atoms are. This result (Figure S1) indicate that calcium-bound all-atom HC is between the X1 and X2 systems.

For control simulations with only water inside the pore, a repulsive potential was used. This potential was generated with a TclBC script routine in NAMD, with a maximal force of 5 kcal/mol at the center of the pore and decreasing to zero at the entrances of the pore.

### 2.3. Permeation Events

Permeation events are calculated from production simulations, extracting the ions and water that cross from one side of the “membrane” to the other, through the central pore. The total number of specific ions or water molecules that permeate this way during all the simulation are divided by the simulation time (20 ns) to obtain the permeation events by nanoseconds. This value is averaged and the standard deviation between the three replicas simulated.

### 2.4. Relative Density

The systems are sampled in sections of 0.5 Å along each axis and the densitity (atoms/Å${}^{3}$) of each particle (potassium, chloride and OH2 atom of water) is extracted from each section using the volmap plugin of VMD. This value is normalized by the area under the curve to obtain a relative density. These values are plotted against *z*-axis values or against radial values (from center of pore going outside) depending on the analysis.

### 2.5. Survival Probability, Dipolar Orientation and Relaxation of Water

The survival probability (SP) of loaded waters was defined as:

(3) $$Q\left(t\right)=\sum_{i=1}^{N}\langle \prod_{t_{k}=t_{0}}^{t_{0}+t}P_{i}\left(t_{k}\right)\rangle$$

$P_{i}$ equals +1 if particle *i* is located inside the channel at time *t* or zero otherwise. Usually, Q(t) decays exponentially with time

(4) $$Q\left(t\right)=A\exp\left(\frac{-t}{\tau_{s}}\right)$$

where $\tau_{s}$ is the half-life time of decay.

Water dipole orientation (WdO) respect to the pore axis i.e., the *z* axis, inside the channels was calculated through the parameter $D_{Z}$, in other words, the collective alignment of the loaded particles along the channel main axis. This parameter is represented by the relation:

(5) $$D_{Z}=\frac{\Sigma \;\mu_{jz}}{\Sigma \;|\vec{\mu_{j}}|}$$

where $\mu_{jz}$ is the z-component of dipole vector and $\vec{\mu_{j}}$ is the dipole vector. $D_{Z}$ will be +1 or −1 if it is fully aligned with *z*-axis, and zero if it is not aligned at all.

At the same time, for the dipole relaxation of loaded waters (DOR), we computed reorientation correlation relaxation functions, $C_{\mu }\left(t\right)$:

(6) $$C_{\mu }\left(t\right)={\langle {\hat{e}}_{\mu }\left(t\right)\cdot {\hat{e}}_{\mu }\left(t_{0}\right)\rangle }_{t_{0}}$$

where ${\hat{e}}_{\mu }$ is a unitary vector pointing along the μ direction, the angular brackets imply average over loaded particles and multiple time origins $C_{\mu }\left(t\right)$, in general, exponentially decays and can be fitted via the following expression:

(7) $$C_{\mu }\left(t\right)=A\exp\left(\frac{-t}{\tau_{\mu }}\right)$$

where $\tau_{\mu }$ stands for first-order rotational relaxation time and *A* is a constant. SP and DOR were calculated from the MDAnalysis *WaterDynamics* module of *python*, as described previously [33].

## 3. Results

### 3.1. Permeation Events

**Figure 2:**
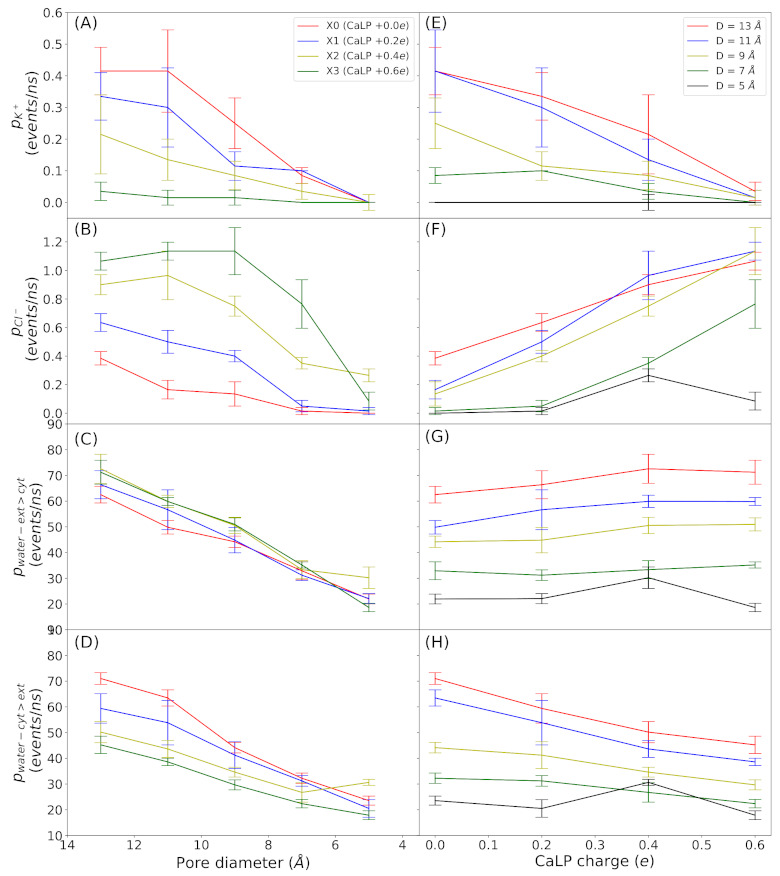
Ion and water permeation events during simulations at V = 500 mV. (**A**,**E**) Potassium permeation. (**B**,**F**) Chloride permeation. (**C**,**G**) Water permeation exterior to interior. (**D**,**H**) Water permeation interior to exterior. In the first column (from **A**–**C**) permeation is plotted against pore diameter, while each color represent a different CaLP charge magnitude. In the second column (from **D**–**F**) permeation is plotted against CaLP charge magnitude, while each color represent a different pore diameter. Legends are indicated in the first plot of each column. All the values are average over three replicas ± SD.

We evaluated the permeation events for potassium, chloride and water molecules through all models at 500 mV, as shown in Figure 2. We test different electric fields to generate different values of voltage across the simulation box (1000 mV and 100 mV) but the results doesn’t change respect to V = 500 mV (Figures S2 and S3). For water, the permeation events are inversely proportional to the pore width and the charge density has no significant effect, even though in some instances there seems to be a inverse relation as the charges is increased (see panels D and H). For both ions, diameters below 7 Å render non-conductive channels. Indeed, for potassium and chloride, the permeation events in the narrowest channel (5 Å) is almost zero, because this value is lower than the effective radius of both ions, this is including the surrounding water shells. Regarding the effect of positive charge density, for diameters above 5 Å chloride and potassium permeation are enhanced and decreased, respectively, as the charge density increases.

Consequently and in term of ions movement, the presence of the CaLP acts as a selectivity filter that allows only the passage of chloride, depending on its charge. In all-atoms simulations of Cx26-HC the presence of calcium in the media and in specific sites inside the pore, render a higher chloride flux [34], similar to what we have observed.

There are studies that show that point charges inside carbon nanotubes (CNTs) can act as selectivity filters for anions or cations [35,36]. In Hilder et al. CNTs are build with carbonyl oxygens at both entrance on the tube, acting as a cationic filter [35]. In this work, when carbonyl oxygen charge is reduced from −0.51e to −0.38e the chloride current raises, in a similar fashion to what is observed in this work. In fact, the cationic nature of Cx26-GJC is also a consequence of surface electrostatics [25].

Additionally to ions, we measure water molecules permeation, attending to simulations done on CNTs, whose have shown that water flux can be altered in nanotubes with point charges inside it [35,36,37,38]. In our systems the permeation of water molecules remains unaltered by the CaLP charge magnitude at least in one sense of the movement, but in the other sense, there is a small decay. In Li et al. the water flow is diminished when a single charge of +1.0e is placed outside the CNT in the plane of the carbon membrane [39]. Probably our channels are wide enough to avoid a more dramatic effect on water flow.

### 3.2. Species Distributions

**Figure 3:**
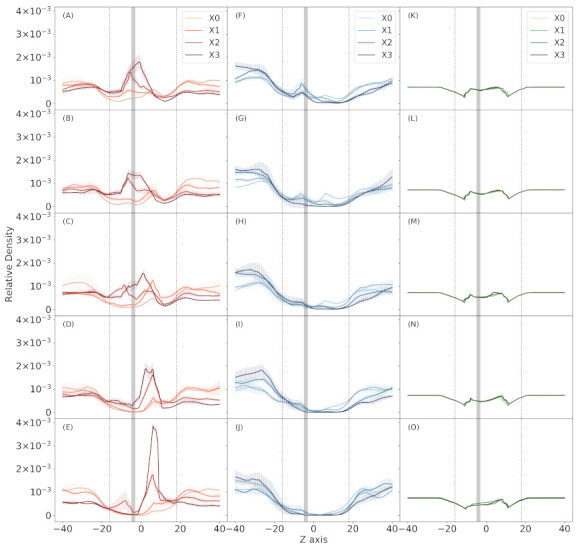
Ion and water distribution across *z*-axis. Red lines (left column) correspond to chloride ion density. Blue lines (center column) correspond to potassium ion density. Green lines (right column) correspond to water molecules density (OH2 atom). Density is normalized against the area under the curve. Each subplot from top to bottom correpond to different pore diameter at the center of the channel in the following order: (**A**,**F**,**K**) 13 Å; (**B**,**G**,**L**) 11 Å; (**C**,**H**,**M**) 9 Å; (**D**,**I**,**N**) 7 Å; and (**E**,**J**,**O**) 5 Å. Each line in each subplot correspond to different CaLP charge magnitude as shown in legend in the top subplot. Solid lines are average over three replicas and shades around lines are SD.

Next, we study the distribution of the solution components across the pore (Figure 3). There is accumulation of chloride around CaLP in wide pore systems with higher CaLP charges. On the other hand, at higher CaLP charges the potassium ions become increasingly excluded from the pore. When the diameter of the pore become narrower chloride ions start to accumulate in the equivalent-to-cytoplasmic side of the pore, not in around the CaLP. In the case of water, there is no effect of CaLP charge, and very little effect of pore diameter, which is more clear in the equivalent-to-extracellular side of the pore.

**Figure 4:**
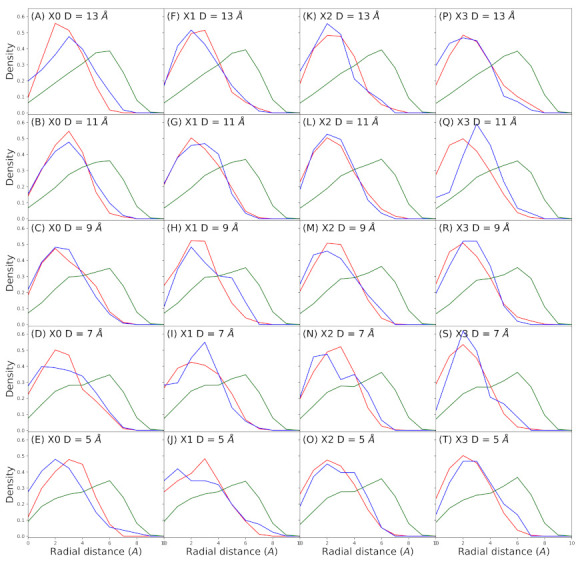
Ion and water radial distribution in all the systems tested. (**A**–**E**) System X0. (**F**–**J**) System X1. (**K**–**O**) System X2. (**P**–**T**) System X3. (**A**,**F**,**K**,**P**) System with 13 Å central diameter. (**B**,**G**,**L**,**Q**) System with 11 Å central diameter. (**C**,**H**,**M**,**R**) System with 9 Å central diameter. (**D**,**I**,**N**,**S**) System with 7 Å central diameter. (**E**,**J**,**O**,**T**) System with 5 Å central diameter. In all plots, red lines is for chloride, blue lines for potassium and green lines for water (OH2 atom). Density is normalized against the area under the curve.

When we study the distribution of the solution components across the pore in function of the pore radius, we see that water and ions are distributed in layers inside the pore (Figure 4), similar to what has been reported for CNTs [38,40,41]. Potassium and chloride tend to be between 3.0 to 4.0 Å from the center of the pore. One interpretation of this distribution is both ions move together, but chloride tend to be closer to pore walls. Water instead tend to be at 6.0 Å from the center, more closely to the pore walls.

Others studies have shown that water arranges in layers inside CNTs, next to the walls, and the number of layers depends on the radius of the pore. Wang et al. had estimated that the optimal diameter to accommodate one and two layer of water is 6.55 and 12.85 Å, respectively [40], but in a system without ions nor applied electric field. Dzubiella et al. also found water layering in system with an electric field generated by ion concentration gradients in two membrane systems [38]. In Peter et al. sodium layer were observed at 2 Å of the center of the pore, next to a water layer that probably supports its solvation layer [41]. In these system, water forms one layer peak between 3 and 4 Å from pore center, depending on pore diameter. Higher electric field allows a second peak around the center of the pore and displaces the other to an outer region. But in these systems there are not ions. The use of a high electric field and the presence of ions could account for that difference in our simulations. Nevertheless it is very interesting to show that even in a very narrow pore the water forms layers, as well as the ions.

In terms of the CaLP charge, this have almost no impact on the radial distribution of the species. The potassium peak is the most variable, but this is correlated with the fact that this ion become progressively excluded from the pore in higher CaLP charges (Figure 3). Water and chloride peaks are invariables when CaLP charge changes, but also in relation the the pore narrowing. At narrower pores the water distribution shows a small shoulder, that correspond to the disturbing of the water layer at the very position of pore narrowing, at the center of the pore.

To explore if the difference in water radial distribution respect to observations in CNTs is due to the presence of ions we run some control simulations. In these simulations a spherical repulsive potential was placed in the middle of the pore excluding only potassium and chloride ions. With this trick we manage to exclude ions completely from the pore but allow water molecules to freely flow. In this systems, water radial distribution is almost the same, only perturbed by the central stretching, but not by the CaLP charge (data not shown). This result indicates that the difference in water radial distribution is caused by the topology of the pore not by the presence of ions in the system. The hexagonal array of carbons characteristic of CNTs but not present in our systems, could also influence the water radial distribution.

### 3.3. Water Dynamics

There are several studies using simulations of CNTs, that shown water molecules could experiment important changes in their dynamic behavior upon application of an electric field and in the presence of point charges into the tube. Because of that we analyze water dynamics using three analysis: survival probability (SP), dipole orientational relaxation (DOR) and dipole orientation along the pore axis (WdO).

**Figure 5:**
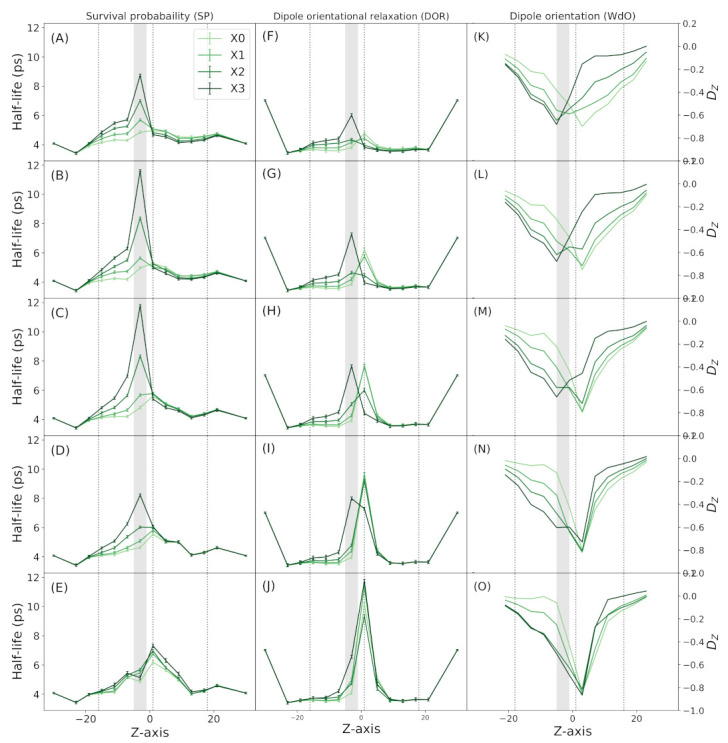
Properties of water molecules during simulations. (**A**–**E**) Half life of decay of survival probability (SP). (**F**–**J**) Half life of decay of dipole orientational relaxation (DOR). (**K**–**O**) Orientation of water dipole ($D_{Z}$) along the pore axis (WdO). (**A**,**F**,**K**) System with 13 Å central diameter. (**B**,**G**,**L**) System with 11 Å central diameter. (**C**,**H**,**M**) System with 9 Å central diameter. (**D**,**I**,**N**) System with 7 Å central diameter. (**E**,**J**,**O**) System with 5 Å central diameter. Dotted lines represent the limits of the channel and the grey shade indicate the position of CaLP. All the values are average over three replicas ± SD when shown.

In terms of SP, we see that water remains more time in the vicinity of CaLP as its charge get higher (Figure 5, left column). When the center of the pore is narrower, this effect is exacerbated, but only to a certain diameter value. The maximum value for water SP around CaLP is in the system X3 with a diameter of 9Å (Figure 5D). In narrower pores, the SP becomes lower, and even in the narrowest pore (5 Å) the effect of CaLP charge is totally abolished. To discard this effect is mediated somehow by the ions, we measure SP in control simulations were ions were excluded from the pore. In these simulation the behaviour of water SP is similar to the normal simulations (Figure S4).

DOR that can be interpreted as the time the water dipole spend in one specific orientation. Higher DOR imply lesser water rotational freedom. In all system simulated, there is a raise in water rotational freedom inside the pore (Figure 5, center column). This rotational freedom is losed around the CaLP position when it have a charge of +0.6. When pore diameter is narrower the rotational freedom also raises, but around the narrow region. In the most narrow pore (Figure 5J) the effect of CaLP is lost, and a very high peak of DOR is observed in the center of the pore.

We also measured the average WdO (Figure 5, right column) along the *z*-axis. In the pores with no CaLP charge there is a clear orientation of the water dipole along the electric field axis in the center of the pore. When the CaLP gets higher the water molecules start to become aligned with the *z*-axis when they are next to CaLPs. When the pore center gets narrower the water gets aligned in the center regardless the charge of CaLP. Previously, a bimodal water orientation has been shown in pores with a point charge at the center, similar to what is observed in aquaporins and other protein channels [37,39,42]. But in these works, the pore have a regular diameter lesser than 11 Å, in contrast with our much wider pores just narrow at the center. Anyway, we are able to observe a bimodal water orientation, in the narrowest pore with the highest CaLP charge, but using a electric field sufficient to generate 1V (data not shown).

All together these analysis indicate that CaLP charge can influence water behavior, specially water SP, but only in channels with an inner diameter of 9 Å or more. In narrower pores, the effect of CaLP is lost and the effects of the pore stretching became dominant.

## 4. Discussion

Molecular dynamics simulation on bioinspired carbon pores is a powerful tool to study the biophysics of biological channels. It offer the possibility of study different biophysical variables in an analytical way. The most usual protein channel modelled using CNTs is the aquaporin [37,39], but also others ionic channels have been mimicked like sodium and calcium channels [36]. Two aspect studied in these CNTs are the transport of single-file water and the presence of selectivity filters. We take advantages from these studies to model an atypical protein channel, much wider and with low selectivity, as the connexin HC. We use carbon-fixed pore to model the protein, instead of CNTs. Connexin channels are affected by voltage differences, but they have irregular inner diameter and multiple charged residues along the pore. In this work we explore the HC-blocking by calcium binding, a widely studied phenomenon, but still without a clear mechanism of action. We explore the two existing hypothesis: an electrostatic effect and a localized pore narrowing.

Fortunately, other authors and ourselves have shown that simple and smaller models can serve to qualitatively asses hypotheses like the electrostatic blocking [25,32,43].

Our results indicate that the pure electrostatic potential elicited by the full binding of calcium ions are not sufficient for block the ionic currents nor the water flux (Figure 2). On the other hand the local pore narrowing should generate a constriction of 5 Å to revent the flux of ions and less than that to stop water flux. We also explore the combination between local pore narrowing and charge presence and we found that at moderate local pore stretching the potassium permeation decreases while the chloride permeation raises, even in the most narrow pore. Instead water flux is only affected by pore narrowing and very marginally by calcium charge.

In all the conditions tested water and ions occupy the pore in layers, from the center of the pore to the walls, where water is close to the walls and ions near but not at the pore center (Figure 4). The radial distributions of ions do not change in any condition but the potassium became excluded from the pore at high CaLP charge magnitude. Instead, radial distribution of water is affected only by the pore narrowing not by the calcium-like charges.

Finally we test three properties of water inside the pore, SP, DOR and WdO (Figure 5). The combined results show that water molecules have a preferential orientation in the middle of the channel. But when they face highly charged CaLP the peak of orientation occurs in the CaLP vicinity, at least in pore with a inner diameter of 9 Å or more.

Going back to connexin channels, the fact crystallographic model of calcium-bound Cx26-GJC is almost identical to calcium-free GJC [22], lead the authors to propose a novel mechanism of calcium blocking where calcium ions form an electrostatic filter to cations in the open-state channel. Nevertheless, others works have rebated this hypothesis. Contreras et al. showed that charged molecules can cross the calcium-binding site in Cx26 HC [23]. Pinto et al, shown that calcium ions stabilize the closed-state of Cx46-HC and in this state, water flux is inhibited. The conclusions of these works are compatible with our results, because bound-calcium by itself is not able to block currents nor water flux, instead a pore stretching is able.

In a 2020 paper, Lee et al. resolved a closed-state Cx31.3 HC where the NTH is obstructung the channel at the cytoplasmic entrance, not at the center of the channel [21]. This is a ground-breaking finding but it is not clear if this mechanism is present in all connexin isoforms.

Most simulations of connexin channels focus on structural aspects of solute transport [44] and the response of the channel to stimuli like voltage difference across the membrane [10,45] or explicit calcium binding [46,47]. The simple system approximation to study biophysical aspects of connexin channels function is promising and more should be done to explore more detailed carbon models or measure properties of solvent in all-atom HC simulations. The solvent properties are attracting attention in different channels as a key element that could explains the transport mechanism, specially in such a complex channels as HCs and GJCs.

## Data Availability

Data available on request from the authors.

## Supplementary Materials

The following are available online at https://www.mdpi.com/article/10.3390/membranes11050372/s1, Figure S1, Effect of CaLP and calcium ions on the electrostatic potential inside the pore, in HC-like and all-atom HC system, respectively; Figure S2, Ion and water permeation events during simulations at V = 1000 mV; Figure S3, Ion and water permeation events during simulations at V = 100 mV; Figure S4. Half life of decay of survival probability in controls simulations where ions cannot enter to the pore.

## Abbreviations

The following abbreviations are used in this manuscript:

HC	Hemichannel
GJC	Gap-junction channel
PH	Parahelix
NTH	N-terminal helix
CaLP	Calcium-like particles
SP	Survival probability
WdO	Water dipole orientation
CNT	Carbon nanotubes
