# Supplementary material for: Simulations on Simple Models of Connexin Hemichannels Indicate That Ca2+ Blocking Is Not a Pure Electrostatic Effect
Source: Membranes (Basel). 2021 May 20;11(5):372. doi: 10.3390/membranes11050372 (PMC8161212; doi:10.3390/membranes11050372)
Supplement: Supplementary file 1 [file membranes-11-00372-s001.zip › membranes-1157868-supplementary.pdf]

Supplemental Material
Ca^{2+} binding does not block ionic currents in Connexin hemichannel by a pure electrostatic effect: a systematic molecular dynamics study

Villanelo F., Carrasco J, Jensen-Flores J, Garate J.A., Perez-acle T.

March 9, 2021

Electrostatic potentials

Average electrostatic potentials from the productions runs, were computed by solving Poisson's equation on a grid employing the *pmepot* plugin of VMD version 1.03 [1] with a grid spacing of 1Å and a Ewald factor of 0.25.

In Figure S1 the electrostatic potentials of different models and the calcium-bound Cx26-HC are presented. When we compare this potential profile with that of the channels used in this work, we see that the all-atom calcium-bound Cx26-HC, generate an electrostatic potential that is between the X1 and X2 models (Figure S1).

References

[1] A. Aksimentiev and K. Schulten, "Imaging alpha-hemolysin with molecular dynamics: ionic conductance, osmotic permeability, and the electrostatic potential map.," *Biophysical journal*, vol. 88, no. 6, pp. 3745–3761.

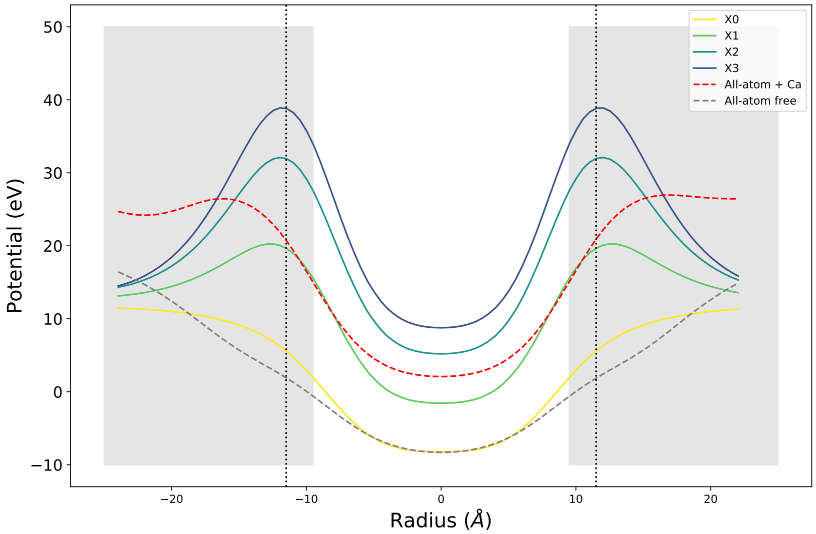

Figure S1: Effect of CaLP and calcium ions on the electrostatic potential inside the pore, in HC-like and all-atom HC system, respectively. The potential shown is radially averaged in the same plane in z-axis where CaLP or calcium ions are (indicated as black dotted lines). Grey shadows show the position of the channel atoms and the white space is the pore lumen.

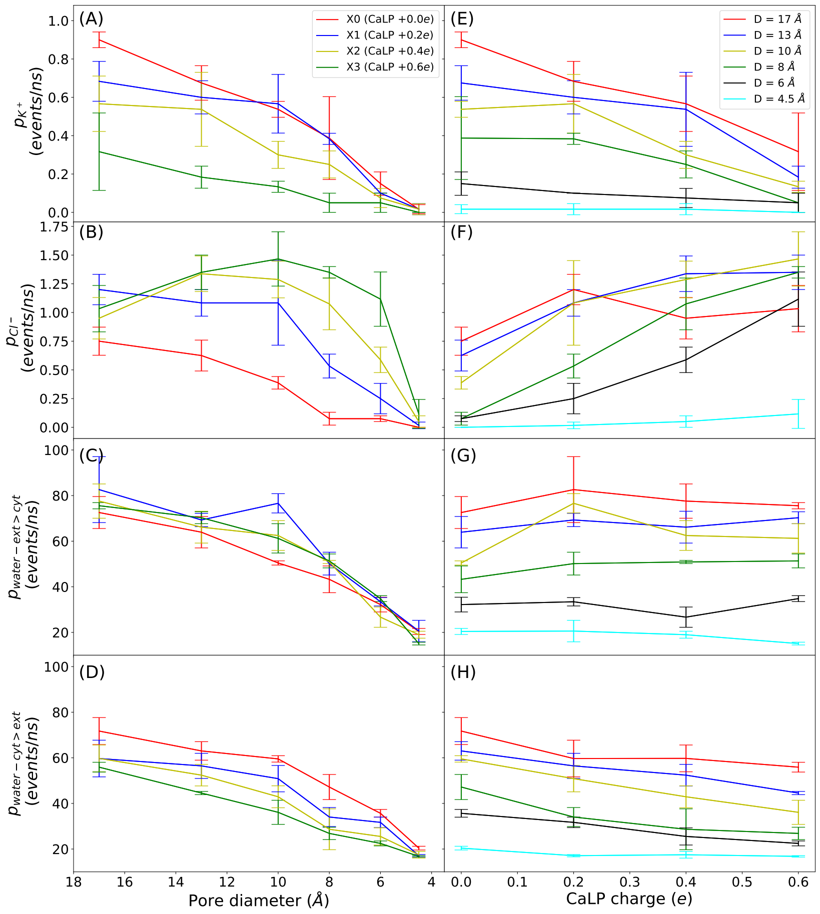

Figure S2: Ion and water permeation events during simulations at V=1000 mV. **A** & **E**. Potassium permeation. **B** & **F**. Chloride permeation. **C** & **G**. Water permeation exterior to interior. **D** & **H**. Water permeation interior to exterior. In the first column (from **A** to **C**) permeation is plotted against pore diameter, while each color represent a different CaLP charge magnitude. In the second column (from **D** to **F**) permeation is plotted against CaLP charge magnitude, while each color represent a different pore diameter. Legends are indicated in the first plot of each column. All the values are average over three replicas ± SD.

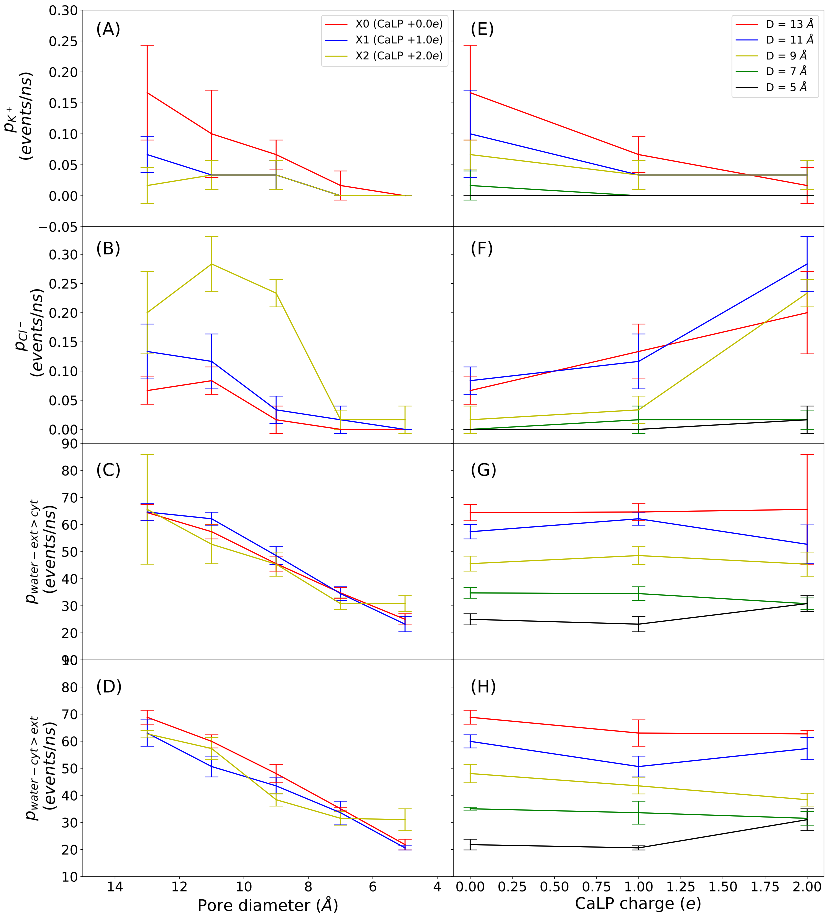

Figure S3: Ion and water permeation events during simulations at V=100 mV. **A & E**. Potassium permeation. **B & F**. Chloride permeation. **C & G**. Water permeation exterior to interior. **D & H**. Water permeation interior to exterior. In the first column (from **A** to **C**) permeation is plotted against pore diameter, while each color represent a different CaLP charge magnitude. In the second column (from **D** to **F**) permeation is plotted against CaLP charge magnitude, while each color represent a different pore diameter. Legends are indicated in the first plot of each column. All the values are average over three replicas ± SD.

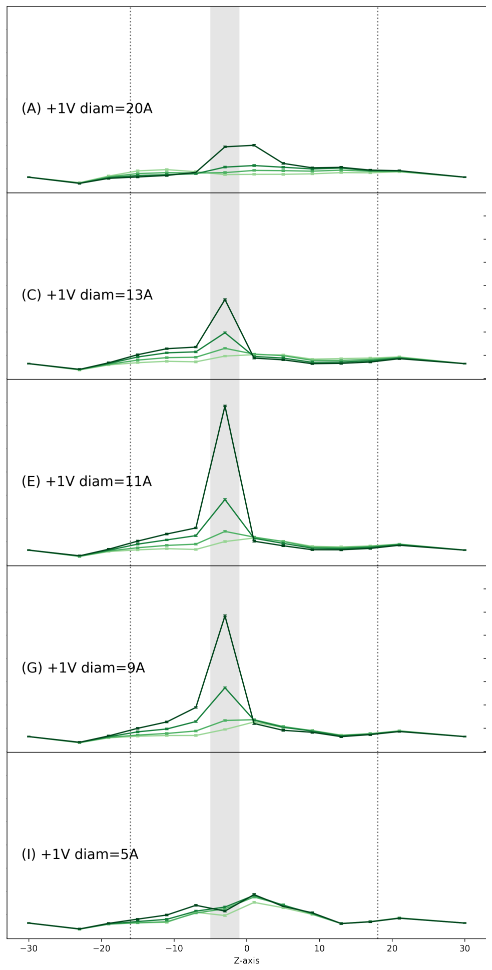

Figure S4: Half life of decay of survival probability in controls simulations where ions cannot enter to the pore. See main text for detail.
